# Predictors of delay seeking delivery services at labor onset among pregnant women in the singida region of Tanzania: analytical cross-sectional study

**DOI:** 10.3389/fgwh.2026.1744192

**Published:** 2026-05-25

**Authors:** Edward Shartiel Nyandala, Joanes Faustine Mboineki

**Affiliations:** Department of Nursing Management and Education, School of Nursing and Public Health, The University of Dodoma, Dodoma, Tanzania

**Keywords:** birth preparedness, delay, knowledge, labor onset, predictors, seeking

## Abstract

**Background:**

Delays in seeking childbirth care are the leading cause of high maternal mortality in developing countries. Despite the Tanzanian government's efforts, such as exempting childbirth services in all government hospitals, timely access to and reporting to health facilities for childbirth care among Tanzanian women remains a challenge.

**Objective:**

The study aimed to assess the predictors of delayed seeking of facility birth at the onset of labor among pregnant women in the Singida region of Tanzania.

**Methodology:**

A hospital-based analytical cross-sectional study was conducted among 384 pregnant women in labor and postpartum women. A principal investigator and research assistants collected the data through a validated questionnaire and checklist. Data were entered and analyzed using the Statistical Package for the Social Sciences (SPSS) software, version 23, through descriptive statistics (frequency and percentages) and inferential statistics [Chi-squared test (*χ*2), and binary logistic regression analysis].

**Results:**

The majority of women 65.1% had type 1 delay, where it took more than one hour to make a decision. Meanwhile, 71.4% women reached the delivery facilities less than one hour, which is type 2 delay, and most of the women 97.1% started receiving care less than thirty minutes after reaching the healthcare facilities. Regarding predictors of delay, type 1 delay was predominant over types 2 and 3, but none of the predictors indicate an association. Most of the women in type 2 delay used less than one hour to reach the facilities, which was influenced by women's residence and religion. Also, the type 3 delay was influenced by women's religion.

**Conclusion:**

Type 1 delay of women making a decision to seek delivery services at healthcare facilities is predominant in the study area. Since sociodemographic characteristics of study participants, delivery services accessibility, and knowledge were found to have no association with type 1 delay, the application of different models and theories should be carried out to investigate specific predictors for type 1 delay in the same participant setting. The type 2 delay is associated with individuals living in rural areas and belonging to the Muslim. Meanwhile, type 3 delay is also influenced by women's religious beliefs.

## Introduction

Ending maternal mortality and improving maternal health have been major global health priorities for decades (1). Approximately 800 women die every day, and 295,000 die annually due to problems associated with pregnancy and delivery worldwide (2, 3). The World Health Organization (WHO) estimates that a woman in a developing country has a 130-fold greater chance of dying from a maternal-related cause during her lifetime than a woman in a developed country (4). For instance, the maternal mortality ratio (MMR) is estimated to be 11 per 100,000 live births in high-income regions and 462 per 100,000 live births in low-income areas (2, 5). Regarding maternal mortality in the developing countries, it accounts for nearly 99% (302,000) of maternal deaths, while Sub-Saharan Africa accounts for around 66% (201,000) of these deaths (6). Sustainable Development Goal 3 (SDG 3) was launched at the United Nations General Assembly in 2015 as part of the ongoing efforts to reduce maternal deaths (2). SDG 3 explicitly targets a global maternal mortality ratio of less than 70 per 100,000 live births by 2030, ensuring that no country has a maternal mortality ratio more than twice the global average (2, 7).

Delays in seeking delivery services at the labor onset in health facilities can potentially lead to obstetric complications that may end up with maternal mortality and morbidity (8). For instance, in developing countries, delays are the leading cause of high maternal mortality (9). It is reported from Northwest Ethiopia that the prevalence of delays in reaching health facilities is 50.6% (10). Another study indicate that, the magnitude of first, second and third maternal delays are 46.80%, 44.00%, and 31.70%, respectively (11). The study conducted in Sierra Leone reported the prevalence of first delay being 23.3% and 26.9% for second delay (12).

The period between the first signs of labor and receiving the first medical attention is referred to as the delay in institutional delivery (9, 13). Three delays have been described; First delay is the time between the onset of labour and the decision to seek emergency obstetric care, which is the leading cause of high maternal deaths in low-income countries (14), second delay refers to the time it takes to get to a health facility after deciding to seek emergency obstetric care, it is a failure to physically arrive to a nearby care facility within an hour of choosing to seek healthcare (15) and the third delay refers to the difficulty in receiving appropriate care within the first five minutes of arriving at the health facility (16). Moreover, another study defined a maternal third delay as the time elapsed between arrival at the hospital and receiving care, which was considered to be 60 min (17).

Some of factors the reported to contribute to the delay in seeking an institutional delivery include: husband's level of education and distance from the healthcare facility (18). Furthermore, mothers' low educational level, unplanned pregnancy, and no nearby health facility are associated with delay (19). Also, family's monthly level of income, mother's decision-making power, mother's parity, mother's knowledge on the importance of delivery to health facility, health facility procedures like episiotomy, and presence of male staff, a few Antenatal care (ANC) visits, mother and husband's occupation are linked to delay (4, 9, 16, 20).

**Figure 1:**
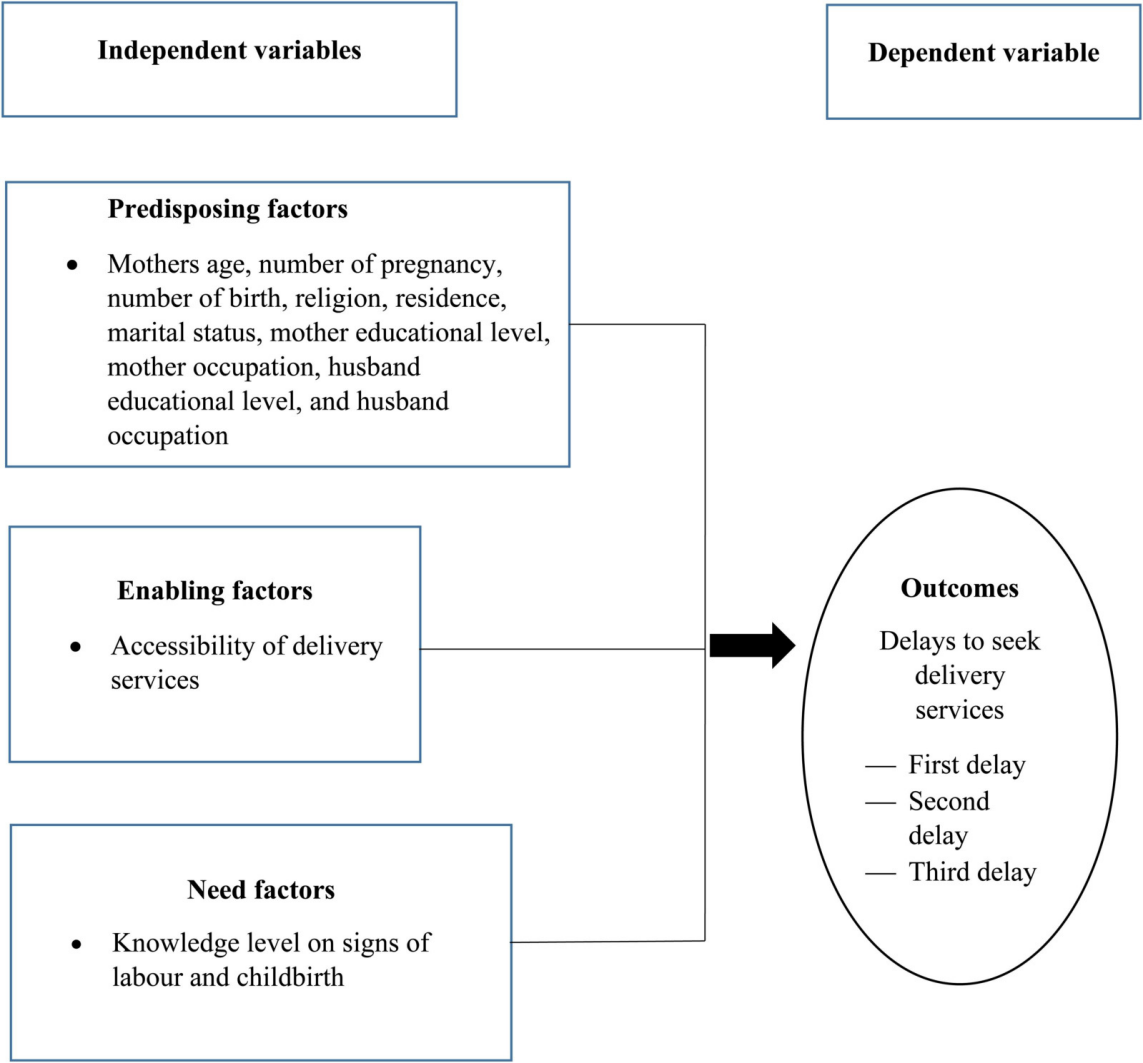
Conceptual framework.

Tanzania is one of ten low-income countries that account for 61% of global maternal deaths (21). Tanzania still has a high maternal mortality rate of 104 maternal deaths per 100,000 live births (22). Due to high maternal mortality, the country did not meet the Millennium Development Goal (MDG) number five (Improving Maternal Health) by 2015 and is now working on the Sustainable Development Goal (SDG) number three, which aims to reduce global maternal mortality to less than 70 per 100,000 live births by 2030 (3, 23). Delay to seeking delivery services at the onset of labour is qualitatively reported to exist in Tanzania and impact quality of care and heightening the demand for obstetric emergencies (24). Despite the government's efforts, such as providing free delivery services in all government hospitals (25) and timely access to services, delay in seeking delivery services remains a challenge (21). Many studies have been conducted in Tanzania to determine the factors contributing to home deliveries; however, few or no studies have specifically examined why pregnant women delay seeking delivery services at a health facility. The current study adopted and integrated Andersen's Behavioral Model and the Three Delays Model. The integration considers the constructs of Andersen's Behavioral Model as predictors (Independent variables) while the constructs of the Three Delays Model to be outcome. Andersen's Behavioral Model includes **predisposing factors**, which involve characteristics that make someone likely to utilize care services, **enabling factors** involving the resources/barriers influencing the utilization of care services, and **need factor** it considers what is perceived to influence utilization of the services (26). With this study, the predisposing factors are the mother's age, number of pregnancies, number of births, religion, residence, marital status, mother's educational level, mother's occupation, husband's educational level, and husband's occupation. Moreover, an enabling factor is the accessibility of delivery services, and a need factor is mothers' knowledge level on signs of labour and childbirth. On the other hand, the Three Delays Model includes three crucial stages that directly affect the survival of both the mother and child: the first delay, the second delay, and the third delay**. The first delay** is linked to family and community factors (27–29), **the second delay** is related to services accessibility (30), and **the third delay** is linked to facility services (28, 29) (Refers to Figure 1).

Therefore, the purpose of this study is to profoundly investigate the predictors of delaying seeking delivery services at the onset of labor among pregnant women. Hence, the dependent variable is the delay in seeking delivery services (first, second, and third delay). In contrast, the independent variables are factors influencing the delay in seeking delivery services (sociodemographic characteristics, knowledge, and accessibility) (Refer to Figure 1). The specific objectives of the study are; (1) To examine the prevalence of delay-seeking delivery services among pregnant women at the onset of labour (2) To examine predictors of delay-seeking delivery services at the onset of labour.

## Methods

### Study area

This study was conducted in the Singida region of Tanzania because of the observed number of pregnant women attending health facilities late for labor, and some of them give birth at home and bring the newborn to a health facility after home delivery. Singida Region is located in the Central Zone of Tanzania, with a total population of 2,008,058 people, comprising 1,012,355 women and 995,703 men (31). Specifically, the study was carried out in a single health center located in the urban area of Singida, named Sokoine Health Center. It is a public local government authority (LGA) facility located in the Misuna area of Singida Municipality, offering primary health services, including reproductive and child health (RCH), to the local community, with government funding supporting improvements and equipment (32).

### Study design

It is a hospital-based cross-sectional study design to investigate the predictors associated with the women's delay in seeking delivery services at the onset of labour.

### Study population

The study considered pregnant women in the latent phase of labor and postnatal women.

### Inclusion and exclusion criteria

All pregnant women in labor, admitted to antenatal wards, and who were willing to participate in the study were recruited. Postpartum women in postnatal wards who were willing to participate in the study were also considered. Pregnant women with obstetric emergencies, unable to express themselves, mentally ill, and unconscious were excluded from the study.

### Sample size determination

The sample size was calculated by using Kish Leslie's formula for quantitative studies (33).

$$\begin{array}{rl} & n=Z^{2}\;P\;(1-P)\\ & D^{2} \end{array}$$

Whereby, *n* = Sample size, D = the standard error in the study, which is 5% P = Proportion of pregnant women delayed seeking delivery service = 50%, Z = the standard normal distribution = 1.96. Therefore, the sample size for this study consisted of 384 women.

### Sampling techniques

A purposive sampling method was employed to select a government hospital in the Singida Urban District of the Singida Region. A convenience sampling technique was used to obtain mothers. The convenience sampling technique was employed to get a representative sample, and it is a method that can provide a satisfactory sample in several situations, despite its potential pitfalls (34).

### Data collection procedure and data collection tool

The data collection was carried out in May 2023 by a principal investigator and two research assistants. The research assistants were trained on research tools and reminded of ethical issues and the modality of data collection. The data were collected through a questionnaire, adapted from the previous study (35) and modified to align with the study objectives. The final English version of the questionnaire had four parts: Part A contained information about the respondents' demographic characteristics, Part B had questions to assess the delay seeking delivery services, Part C contained information about the accessibility of health care delivery services, and Part D contained information on respondents' knowledge about signs of labour and childbirth. The final version of the English questionnaire was translated into the Swahili language to facilitate participants' understanding. The data were collected during the daytime for convenience and to enhance full participation from the study participants.

### Data analysis

Data were entered and analyzed using the Statistical Package for Social Sciences (SPSS) software, version 23. The respondents' sociodemographic characteristics were analyzed using descriptive statistics, including frequency and percentages. Furthermore, since some of the variables, such as accessibility of delivery services and knowledge level of true signs of labor and childbirth, had multiple items as indicators, the overall frequency and percentages were obtained by considering participants with equal or above the mean score to rank high compared to those having below the mean score. Specifically, the delay seeking delivery services was assessed with three items to capture type 1, 2, and 3 delays.

The association between variables was determined through a cross-tabulation, Chi-squared test (*χ*2), and *P*-value. Univariate logistic regression analysis was used to compute the magnitude of association between social-demographic characteristics and all statistically significant types of delay in seeking delivery services at the onset of labor. Also, Binary logistic regression analysis were used to examine the magnitude of association between accessibility of delivery services and all statistically significant types of delay. The binary logistic regression was an appropriate model in this study as the dependent variables had two levels each, for instance, type 1 delay (<1 h and >1 h), type 2 delay (<1 h and >1 h), and type 3 delay (<30 min and >30 min). Meanwhile, as the model requires at least one independent variable to predict the dependent, the study had three predictors of types of delays, such as sociodemographic, knowledge of the signs of labour and childbirth, and accessibility of healthcare delivery services.

## Ethics approval and consent to participate

The ethical approval to conduct this study was obtained from the University of Dodoma Institutional Research Review Committee (UDOM-IRREC) before the data collection procedure, with identification number MA.84/261/01/62/333. Permission to conduct this study at the particular health facility was obtained from the Medical Officer in charge of Sokoine Health Center. Informed consent was obtained from the respondents. For participants below 18 years of age, their guardians were asked to complete the informed consent on their behalf. The confidentiality of participants' information was ensured by avoiding the use of participants' names and keeping all completed questionnaires out of the hands of non-research team members. Participants were free to participate in and withdraw from the study at any time they felt it was appropriate to do so.

## Results

### Sociodemographic characteristics of the participants

The mean age of mother was Mean ± SD (Min-Max) 26.19 ± 6.19 (17–41), whereby the majority of them belonged to the age group of 15–24 years, 180 (46.9%). They had more than one pregnancy, 213 (55.5%), with Mean ± SD (Min-Max) 2.42 ± 1.73 (1–8), and had delivered more than once, 200 (52.1%), with Mean ± SD (Min-Max) 2.21 ± 1.57 (0–8). Participants were Christian 194 (50.5%) and Muslim 190 (49.5%), and most of them lived in urban areas 202 (52.6%). They were married 310 (80.7%), had a secondary level of education 185 (48.2%), and were unemployed 325 (84.6%). Their husbands had a secondary level of education, 174 (45.3%), and were not employed, 282 (73.4%) (Refer to Table 1).

**Table 1:** Sociodemographic characteristics of the study participants (*n* = 384).

Variables	Frequency (*n*)	Percentage (%)
Mothers age Mean ± SD (Min-Max) 26.19 ± 6.19 (17–41)		
15–24	180	46.9
25–34	153	39.8
35–49	51	13.3
Number of pregnancies Mean ± SD (Min-Max) 2.42 ± 1.73 (1–8)		
One pregnancy	171	44.5
More than one pregnancy	213	55.5
Number of births Mean ± SD (Min-Max) 2.21 ± 1.57 (0–8)		
One birth	184	47.9
Multiple births	200	52.1
Religion		
Christian	194	50.5
Muslim	190	49.5
Residence		
Rural area	182	47.4
Urban area	202	52.6
Marital status		
Single	74	19.3
Married	310	80.7
Mothers educational level		
Never attend school	20	5.2
Primary school	123	32.0
Secondary school	185	48.2
Vocational training school	16	4.2
College or University	40	10.4
Husband educational level		
Never attend school	26	6.8
Primary school	96	25.0
Secondary school	174	45.3
Vocational training school	45	11.7
College or University	43	11.2
Mothers’ occupation		
Unemployed	325	84.6
Employed	59	15.4
Husband occupation		
Unemployed	282	73.4
Employed	102	26.6

### Prevalence of delay-seeking delivery services among pregnant women at the onset of labour

Regarding type 1 delay, which is the time taken to decide to seek emergency obstetric care, most of the participants, 250 (65.1%), spent more than one hour to make a decision. About type 2 delay, which is concerned with the time taken by a woman to get to the facility, the majority of participants, 274 (71.4%), used less than one hour to reach the facilities. For type 3 delay, which involves the time taken to receive care at the facility, most of the participants, 373 (97.1%), reported having started receiving care less than thirty minutes after reaching the healthcare facilities (Refer to Table 2).

**Table 2:** Delays in seeking delivery services at the onset of labour (*n* = 384).

Type of delay	Frequency (*n*)	Percentage (%)
Type 1 delay		
<1 h	134	34.9
>1 h	250	65.1
Type 2 delay		
<1 h	274	71.4
>1 h	110	28.6
Type 3 delay		
<30 min	373	97.1
>30 min	11	2.9

### Predictor of delay in seeking birth at health facility

Regarding the **first delay**, the Chi-squared test was performed to identify the association between sociodemographic factors and the type 1 delay in seeking delivery services at the onset of labor. None of the sociodemographic characteristics was significantly associated with type 1 delay of making a decision after the onset of labour (Refer to Table 3). Regarding the **second delay**, the Chi-squared test identified a significant association between social-demographic characteristics and a second delay in seeking delivery services at the onset of labour in two variables: religion (*χ*2 = 3.62, *P* = 0.05) and residence (*χ*2 = 51.89, *P* < 0.01) (Refer to Table 4). In binary logistic regression, participants who lived in rural areas were 5.8 (OR) times more likely to delay reaching the delivery facilities (*P* < 0.001; 95% CI: 3.503, 9.609). Also, Participants who belonged to the Christian religion were 1.54 (OR) times more likely to delay reaching the delivery facilities (*P* = 0.05; 95% CI: 0.986, 2.41) (Refer to Table 5). Regarding the **third delay**, only one sociodemographic variable, which is the religion, was significantly associated with type 3 delay (*χ*2 = 7.78, *P* = 0.01). Other sociodemographic characteristics were not significantly associated with type 3 delay (Refer to Table 6).

**Table 3:** Sociodemographic characteristics associated with type 1 delay.

Variables	Type 1 delay		*χ*2	*P*
	Below 1 h *n* (%)	Above 1 h *n* (%)		
Mothers age				
15–24	69 (51.5)	111 (44.4)	2.98	0.23
25–34	52 (38.8)	101 (40.4)		
35–49	13 (9.7)	38 (15.2)		
Number of pregnancies				
One pregnancy	66 (49.3)	105 (42)	1.86	0.17
More than one pregnancy	68 (50.7)	145 (58)		
Number of births				
One birth	69 (51.5)	115 (46)	1.06	0.3
Multiple births	65 (48.5)	135 (54)		
Religion				
Christian	67 (50)	127 (50.8)	0.02	0.88
Muslim	67 (50)	123 (49.2)		
Residence				
Rural area	61 (45.5)	121 (48.4)	0.29	0.59
Urban area	73 (54.5)	129 (51.6)		
Marital status				
Single	31 (23.1)	43 (17.2)	1.98	0.16
Married	103 (76.9)	207 (82.8)		
Mothers educational level				
Never attend school	8 (6)	12 (4.8)	5.45	0.24
Primary school	46 (34.3)	77 (30.8)		
Secondary school	68 (50.7)	117 (46.8)		
Vocational training school	4 (3)	12 (4.8)		
College or University	8 (6)	32 (12.8)		
Husband educational level				
Never attend school	7 (5.2)	19 (7.6)	7.46	0.11
Primary school	38 (28.4)	58 (23.2)		
Secondary school	67 (50)	107 (42.8)		
Vocational training school	9 (6.7)	36 (14.4)		
College or University	13 (9.7)	30 (12)		
Mothers' occupation				
Unemployed	119 (88.8)	206 (82.4)	2.75	0.09
Employed	15 (11.2)	44 (17.6)		
Husband occupation				
Unemployed	106 (79.1)	176 (70.4)	3.38	0.07
Employed	28 (20.9)	74 (29.6)		

**Table 4:** Sociodemographic characteristics associated with type 2 delay.

Variables	Type 2 delay		χ2	*P*
	Below 1 h *n* (%)	Above 1 h *n* (%)		
Mothers age				
15–24	127 (46.4)	53 (48.2)	0.75	0.69
25–34	108 (39.4)	45 (40.9)		
35–49	39 (14.2)	12 (10.9)		
Number of pregnancies				
One pregnancy	117 (42.7)	54 (49.1)	1.29	0.26
More than one pregnancy	157 (57.3)	56 (50.9)		
Number of births				
One birth	128 (46.7)	56 (50.9)	0.55	0.46
Multiple births	146 (53.3)	54 (49.1)		
Religion				
Christian	130 (47.4)	64 (58.2)	3.62	0.05*
Muslim	144 (52.6)	46 (41.8)		
Residence				
Rural area	98 (35.8)	84 (76.4)	51.89	<0.01*
Urban area	176 (64.2)	26 (23.6)		
Marital status				
Single	48 (17.5)	26 (23.6)	1.89	0.17
Married	226 (82.5)	84 (76.4)		
Mothers educational level				
Never attend school	9 (3.3)	11 (10)	8.6	0.07
Primary school	85 (31)	38 (34.5)		
Secondary school	137 (50)	48 (43.6)		
Vocational training school	12 (4.4)	4 (3.6)		
College or University	31 (11.3)	9 (8.2)		
Husband educational level				
Never attend school	15 (5.5)	11 (10)	7.79	0.1
Primary school	64 (23.4)	32 (29.1)		
Secondary school	124 (45.3)	50 (45.5)		
Vocational training school	38 (13.9)	7 (6.4)		
College or University	33 (12)	10 (9.1)		
Mothers’ occupation				
Unemployed	233 (85)	92 (83.6)	0.12	0.73
Employed	41 (15)	18 (16.4)		
Husband occupation				
Unemployed	195 (71.2)	87 (79.1)	2.53	0.11
Employed	79 (28.8)	23 (20.9)		

* Significant difference.

**Table 5:** The extent of association of predictors on type 2 delay in reaching the delivery facility.

Variable	OR	*P*-value	95% CI	
			Low	Upp
Residence				
Rural area	5.8	<0.001	3.503	9.609
Urban area (Ref)	-	-	-	-
Religion				
Christian	1.54	0.05	0.986	2.41
Muslim (Ref)	-	-	-	-
Accessibility of delivery services				
No accessibility	0.01	<0.001	0.004	0.028
There is accessibility (Ref)	-		-	-

**Table 6:** Sociodemographic characteristics associated with type 3 delay.

Variables	Type 3 delay		χ2	*P*
	Below 30 min	Above 30 min		
	*n* (%)	*n* (%)		
Mothers age				
15–24	176 (47.2)	4 (36.4)	0.56	0.76
25–34	148 (39.7)	5 (45.5)		
35–49	49 (13.1)	2 (18.2)		
Number of pregnancies				
One pregnancy	167 (44.8)	4 (36.4)	0.31	0.58
More than one pregnancy	206 (55.2)	7 (63.6)		
Number of births				
One birth	180 (48.3)	4 (36.4)	0.61	0.44
Multiple births	193 (51.7)	7 (63.6)		
Religion				
Christian	193 (51.7)	1 (9.1)	7.78	0.01*
Muslim	180 (48.3)	10 (90.9)		
Residence				
Rural area	175 (46.9)	7 (63.6)	1.19	0.27
Urban area	198 (53.1)	4 (36.4)		
Marital status				
Single	72 (19.3)	2 (18.2)	0.01	0.93
Married	301 (80.7)	9 (81.8)		
Mothers educational level				
Never attend school	20 (5.4)	0 (0)	3.08	0.54
Primary school	118 (31.6)	5 (45.5)		
Secondary school	180 (48.3)	5 (45.5)		
Vocational training school	15 (4)	1 (9.1)		
College or University	40 (10.7)	0 (0)		
Husband educational level				
Never attend school	23 (6.2)	3 (27.3)	7.55	0.11
Primary school	94 (25.2)	2 (18.2)		
Secondary school	170 (45.6)	4 (36.4)		
Vocational training school	44 (11.8)	1 (9.1)		
College or University	42 (11.3)	1 (9.1)		
Mothers’ occupation				
Unemployed	316 (84.7)	9 (81.8)	0.07	0.79
Employed	57 (15.3)	2 (18.2)		
Husband occupation				
Unemployed	273 (73.2)	9 (81.8)	0.41	0.52
Employed	100 (26.8)	2 (18.2)		

* Significant difference.

### Knowledge level of the true signs of labour and childbirth among pregnant women and its association with delayed seeking healthcare services

**Figure 2:**
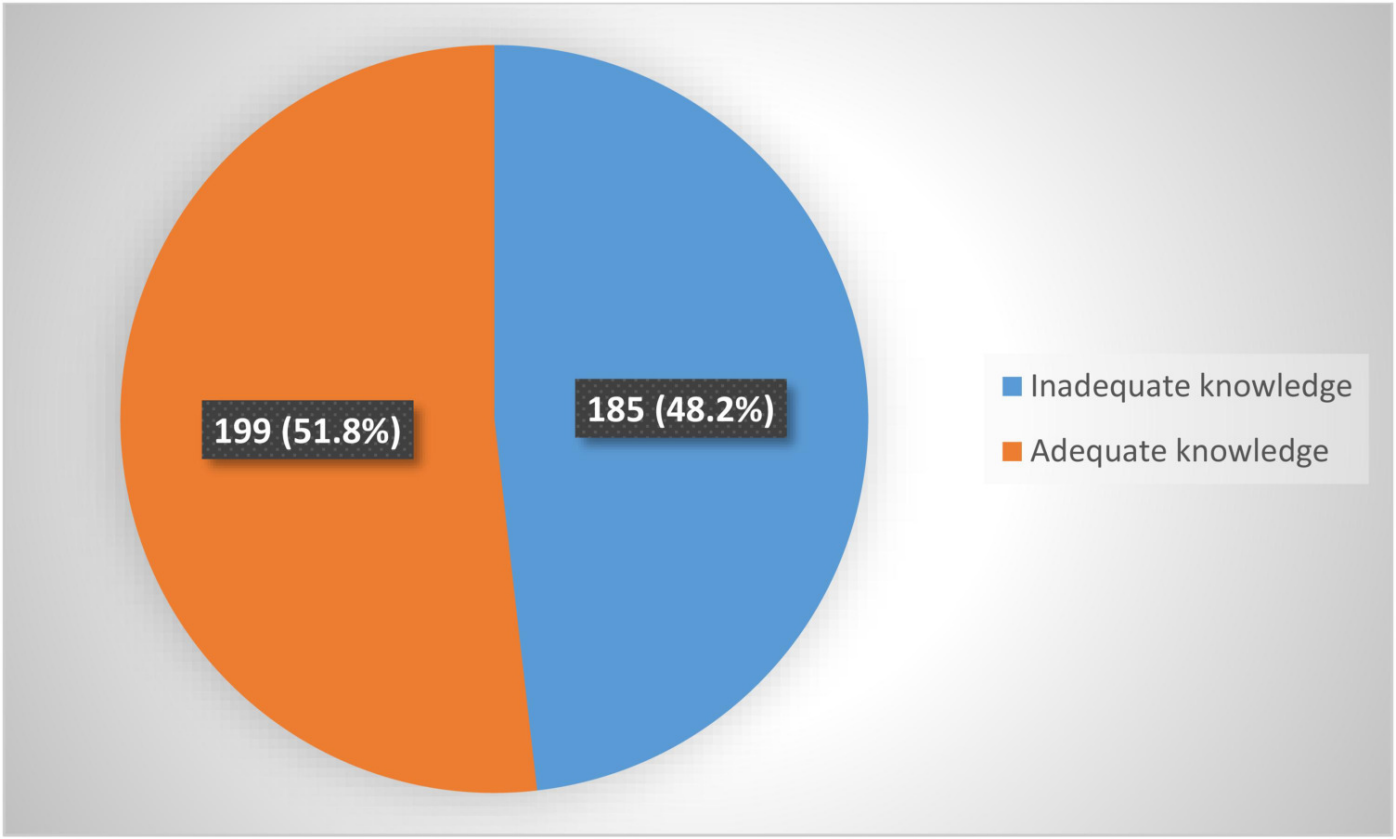
Knowledge level of the signs of labour and childbirth.

In item analysis (Table 7), 155 (40.4%) of participants agreed that contractions or tightening were a sign of labor and childbirth. The majority of women in three items strongly agreed that the presence of bloody or reddish mucus discharge from the vagina 142 (37%), abdominal and lower back pain that does not go away 185 (48.2%), and breaking of water 184 (47.9%) are the signs of labour and childbirth. However, in one item, women were neutral when asked about the signs of labour and childbirth, 138 (35.9%) (Refer to Table 6). The overall knowledge level among participants was computed through descriptive statistics, with mean score indicated as Mean ± SD (Min-Max) 19.14 ± D.8 2 (11–25). Therefore, overall, most of the participants, 199 (51.8%), were found to have sufficient knowledge of the signs of labour and childbirth (Refer to Figure 2). Regarding the influence of knowledge level on delay seeking delivery services, there was no significant association of knowledge level with any type of delay (Refer to Table 8).

**Table 7:** Item analysis for knowledge of the signs of labour and childbirth.

Items	Frequency (*n*)	Percentages (%)
Presence of Contractions or tightening		
Strongly disagree	2	.5
Disagree	86	22.4
Neutral	71	18.5
Agree	155	40.4
Strongly agree	70	18.2
Presence of bloody or reddish mucus discharge from the vagina		
Strongly disagree	-	-
Disagree	28	7.3
Neutral	73	19.0
Agree	141	36.7
Strongly agree	142	37.0
Abdominal and lower back pain that does not go away		
Strongly disagree	1	.3
Disagree	23	6.0
Neutral	19	4.9
Agree	156	40.6
Strongly agree	185	48.2
Breaking of water		
Strongly disagree	2	.5
Disagree	31	8.1
Neutral	62	16.1
Agree	105	27.3
Strongly agree	184	47.9
Feeling the urge to go to the toilet		
Strongly disagree	20	5.2
Disagree	89	23.2
Neutral	138	35.9
Agree	95	24.7
Strongly agree	42	10.9

**Table 8:** Association of knowledge level and types of delays.

Variable	Knowledge level		χ^2^	*P*
	Adequate knowledge level *n* (%)	Inadequate knowledge level *n* (%)		
Type 1 delay				
<1 h	66 (49.3)	68 (50.7)	0.54	0.46
>1 h	133 (53.2)	117 (46.8)		
Type 2 delay				
<1 h	142 (51.8)	132 (48.2)	0.0	0.99
>1 h	57 (51.8)	53 (48.2)		
Type 3 delay				
<30 min	193 (51.7)	180 (48.3)	0.03	0.86
>30 min	6 (54.5)	5 (45.5)		

### Accessibility of healthcare delivery services at the onset of labour

The accessibility of healthcare delivery services at the onset of labour was measured using six items: availability, affordability, type of transportation used, road quality, distance, and time taken to travel to the health facility. The findings revealed that 279 (72.7%) of respondents took a short time to get transport, 331 (86.2%) had quality roads, and 225 (58.6%) had a nearby healthcare facility for delivery services. Moreover, 274 (71.4%) of participants took less than one hour to travel to the facility, 384 (100%) had means of transport, and 262 (68.2%) of participants reported that transport was not costly (Refer to Table 9). Overall, most of the participants, 221 (57.6%), had good accessibility of healthcare delivery services at the onset of labour. Meanwhile, in the Chi-squared test, accessibility was significantly associated with only type 2 delay, which is concerning women to reach to hospital less than one hour after the onset of labor (*χ*^2^ = 183.44, *P* < 0.001) (Refer to Table 10). In binary logistic regression, participants who had no accessibility of delivery services were 0.01 (OR) times less likely to delay reaching the delivery facilities (*P* < 0.001; 95% CI: 0.004, 0.028) (Refer to Table 5).

**Table 9:** Accessibility of healthcare delivery services at the onset of labour.

Variable	Number (n)	Percentage (%)
Time taken to get transport to travel to a nearby hospital		
<1 h	279	72.7
>1 h	105	27.3
Quality of road used while traveling to a health facility		
Good road	331	86.2
Poor road	53	13.8
Distance to the healthcare facility		
<5 km	225	58.6
>5 km	159	41.4
Time taken to travel and reach a health facility		
<1 h	274	71.4
>1 h	110	28.6
Type of transport		
Have no means of transport	-	-
Have means of transport	384	100
Was the type of transport used cost		
Yes	122	31.8
No	262	68.2

**Table 10:** Association of accessibility of healthcare delivery services at the onset of labour and types of delay.

Variable	Accessibility of delivery services		χ^2^	*P*
	There is accessibility *n* (%)	No accessibility *n* (%)		
Type 1 delay				
<1 h	77 (34.8)	57 (35)	0.001	0.98
>1 h	144 (65.2)	106 (65)		
Type 2 delay				
<1 h	217 (79.2)	57 (20.8)	183.44	<0.001*
>1 h	4 (3.6)	106 (96.4)		
Type 3 delay				
<30 min	217 (58.2)	156 (41.8)	2.08	0.15
>30 min	4 (36.4)	7 (63.6)		

* A value indicating a statistically significant association for the particular variable.

## Discussion

### Types of delays in seeking delivery services

It was found that many women 65.1% had type 1 delay, where it took more than one hour to make a decision. Meanwhile, 71.4% women had type 2 delay, where they reached the delivery facilities less than one hour. Most of the women 97.1% had type 3 delay, where they started receiving care less than thirty minutes after reaching the healthcare facilities. The findings of the current study are consistent with findings of previous studies (8, 36), reported type 1 delay a leading one, whereby 32% of women waited more than 72 h from symptom onset to deciding to seek clinical care (37).

### Predictors of delay in seeking delivery services

Even though type 1 delay was predominant over type 2 and 3, none of the predictors indicates that it influences it. The studied predictors did not show their association with type 1 delay because of the nature of the population and community setting. There should be unidentified and unstudied predictors that are specific reasons for type 1 delay. The previous literature reported that despite distances to health facilities, high cost, and negative past experiences perceived to be common for type 1 delay, but in most communities, they are suppressed and dominated by women's perceived severity of illness (27). In contrast, this study found most of the women in type 2 delay used less than one hour to reach the facilities, which was influenced by women's' residence and the religion they belong. Women who lived in rural areas were times more likely to delay reaching the delivery facilities, this is because of the long distance to the delivery facilities and high cost, especially for transport fare and for eating on the way. Being in a rural area, it is difficult to get means of transport, which is why some women decide to walk on foot to the facilities. It is supported by other studies showing that individuals residing in remote areas have the absence of a health facility within a 30 min distance of walking that's why they end up with home deliveries (38, 39). Meanwhile, for type 2 delay, women who belonged to the Christian religion were times more likely to delay reaching the delivery facilities compared to their counterpart Muslim women. This could be attributed by lack of health awareness sessions carried out in churches, in contrast to the Mosques that might have religious teachings linked to health issues, making women avoid delaying. Some of the religious houses invite healthcare professionals to communicate issues related to health. The literature reported that educational programs tailored to Muslim beliefs improve mental and physical health outcomes and promote positive health behaviors (40).

### Study limitation

The studied predictors for delays were very few that limited a broader investigation on what influences types of delays. Also, the studied predictors were not derived from a baseline qualitative study; rather, it was based on the existing literature that would have excluded the realistic predictors in the local context. The sample size used isn't sufficient, as a larger sample could more accurately represent the population and be better suited to produce strong and reliable results. The data collection was carried out during the day, automatically excluding those delivered at night, which is a source of bias in obtaining participants. This facility-based study might have introduced selection bias because the primary outcome is delay in seeking/reaching care. Women who delivered at home or on the way were systematically excluded, likely underestimating both first and second delays. Furthermore, the use of convenience sampling limits the study as it is subject to sampling bias and systematic errors. Social desirability bias could have occurred since some variables were assessed through self-reporting, such as accessibility of delivery services. The study excluded women with emergency obstetrics, who might be among those who are affected by the delay. Finally, only univariate logistic regression findings are reported, that limit the understanding of extent of interaction between independent and dependent variables.

## Conclusion

Type 1 delay of women making a decision to seek delivery services at healthcare facilities is predominant in the study area. However, none of the sociodemographic characteristics, and participants' knowledge level, nor accessibility of delivery services were significantly associated with it. Since sociodemographic characteristics of study participants, delivery services accessibility, and knowledge were found to have no association with type 1 delay, the application of different models and theories should be carried out to investigate specific predictors for type 1 delay in the same participant setting. The type 2 delay is associated with individuals living in rural areas and belonging to the Muslim. Meanwhile, type 3 delay is also influenced by women's religious beliefs. For type 2 and 3 delays, the implementation of health education through religious houses needs to continue, and the building of healthcare facilities near people should be a priority.

## Data Availability

The original contributions presented in the study are included in the article/Supplementary Material, further inquiries can be directed to the corresponding author.
