# Supplementary material for: Predictors of delay seeking delivery services at labor onset among pregnant women in the singida region of Tanzania: analytical cross-sectional study
Source: Front Glob Womens Health. 2026 May 25;7:1744192. doi: 10.3389/fgwh.2026.1744192 (PMC13243385; doi:10.3389/fgwh.2026.1744192)
Supplement: Supplementary file 1 [file Supplementaryfile1.pdf]

Supplementary material

Questionnaire (English version)
This questionnaire will assess the predictors of delay seeking delivery services at the onset of labour.

IDENTIFICATION

Name of Ward ………………… ….. Name of the Street…………………..

Participant ID code..……. …….. …. Name of hospital ……………………

Researcher name…………………….

Date of interview...............................

PART A: Social-demographic and obstetric characteristics

	QUESTION	RESPONSE
1.1	How old are you**?**	Age in years……….
1.2	How many pregnancies have you ever had?	Number…………..
1.3	How many times did you give birth?	Number…………..
1.4	What is your religion? (PUT a tick on the appropriate response)	a) Christian<br>b) Muslim<br>c) Others
1.5	What is your residence? (PUT a tick on the appropriate response)	a) Rural<br>b) Urban
1.6	What is your current marital status? (PUT a tick on the appropriate response)	a) Single<br>b) Married or living together<br>c) Separated/ Divorced<br>d) Widow
1.7	What is the highest level of education you attended? (PUT a tick on the appropriate response)	a) primary<br>b) Secondary<br>c) Vocation training<br>d) College/University<br>e) Never attended
1.8	What is the highest level of education attained by your husband? (PUT a tick on the appropriate response)	a) primary<br>b) Secondary<br>c) Vocation training<br>d) College/University<br>e) Never attended
1.9	What is your current occupation? (PUT a tick on the appropriate response)	a) Employed<br>b) Unemployed<br>c) Housewife

1.10	What is your husband's current occupation? Economic status of your husband? (PUT a tick on the appropriate response)	a) Employed<br>b) Unemployed
1.11	Who made decided whether or not to go to a health facility for assistance? (PUT a tick on the appropriate response)	a) Respondent (mother)<br>b) Husband<br>c) Respondent and husband<br>d) Other family members
1.12	What is your average earning per month (PUT a tick on the appropriate response)	a) Above Tshs 138000<br>b) Tshs 69000-138000<br>c) Below Tshs 69000
1.13	Did you attend antenatal care visits? (PUT a tick on the appropriate response)	a) Yes<br>b) No
1.14	How many antenatal care visits did you attend? (PUT a tick on the appropriate response)	a) Less than 4 visits<br>b) Above 4 visits
1.15	At what time you had already arrived at the health facility? (PUT a tick on the appropriate response)	a) Less than 30 minutes<br>b) Between 30-60 minutes<br>c) Above 1 hour

PART B: Delay Seeking Delivery Services at the Onset of Labour

Questions	Responses
**Question on the first delay:** How long did it take to decide to go to the health facility after start feeling labour signs and symptoms?	a) less than 30 minutes<br>b) Between 30-60 minutes<br>c) Above 1 hour
**Question for the second delay:** How long did it take to travel and reach the health facility?	a) Less than 30 minutes<br>b) Between 30-60 minutes<br>c) Above 1 hour
**Question for Third delay:** How long did it take to start receiving care after arriving at a health facility? "	a) Less than 30 minutes<br>b) Between 30-60 minutes<br>c) Above 1 hour

PART C: Accessibility of healthcare delivery services

	QUESTION	RESPONSE
2.1	How long did it take to find and get transport once a decision was made to go to the health facility? (PUT a tick on the appropriate response)	a) Less than 1 hour<br>b) Above 1 hour
2.2	What is the quality of the road that you used when traveling to a health facility? (PUT a tick on the appropriate response)	a) Poor<br>b) Very poor<br>c) Moderate<br>d) Good<br>e) Very good
2.3	What is the estimated distance from your home to the nearby health facility? ?(PUT a tick on the appropriate response)	a) Less than 5 km<br>b) Above 5 km
2.4	How long did it take to travel and reach the health facility? (PUT a tick on the appropriate response)	a) Less than 1 hour<br>b) Above 1 hour
2.5	Which means of transport did you use to travel to the health facility? (PUT a tick on the appropriate response)	a) Car/motorcycle<br>b) Public transport (bus/tax)<br>c) Walking<br>d) Bicycle<br>e) Others
2.6	Were the means of transport used to cost you based on the amount of money you had? (PUT a tick on the appropriate response)	a) Yes<br>b) No

PART D: Knowledge of signs of labour and childbirth

4.1	Do you know the signs of labour and childbirth? (PUT a tick on the appropriate response)	a) Yes<br>b) No				
4.2	Are the following signs of childbirth? Put a tick in a box against each statement according to the scale below					
	Contractions or tightening	Strongly disagree<br>1	Disagree<br>2	Neutral<br>3	Agree<br>4	Strongly agree<br>5
	Presence of bloody or reddish mucus discharge from the vagina					
	Belly and lower back pain that does not go away					
	Breaking of water					
	Feeling the urge to go to the toilet					
4.3	Signs of labour can make you delay seeking delivery services and increase the risk of death due to pregnancy					
